# Emergency Department Utilization by Patients With Eating Disorders: A National Population-Based Study

**DOI:** 10.7759/cureus.28526

**Published:** 2022-08-29

**Authors:** Precious Eseaton, Eseosa Sanwo, Solomon O Anighoro, Eboma John, Nelson O Okobia, Uaiye Enosolease, Rebecca E Enejo, Ehizogie Edigin

**Affiliations:** 1 College of Medicine, University of Benin, Benin City, NGA; 2 General Practice, St. Helens and Knowsley Teaching Hospitals NHS Trust, Whiston, GBR; 3 Department of Internal Medicine, University of Benin/General Hospital Kazaure, Kazaure, NGA; 4 Department of Surgery, Federal Medical Center, Lokoja, NGA; 5 Department of Internal Medicine, John H. Stroger, Jr. Hospital of Cook County, Chicago, USA

**Keywords:** high-income household, major depressive disorder, nationwide emergency department sample, emergency department, eating disorder

## Abstract

Background

There is a scarcity of national United States (U.S) data on emergency department (ED) utilization by patients with eating disorders. This study aims to determine the most common reasons for ED visits of patients with eating disorders, as well as baseline characteristics of patients who present due to eating disorders.

Methods

We obtained data from the Nationwide Emergency Department Sample (NEDS), the largest all-payer ED database in the United States. Each ED visit in NEDS 2018 can have only one "principal" diagnosis, which is the main reason for the visit and up to 34 "secondary" diagnoses. We abstracted data for all ED visits with "any" diagnosis of an eating disorder, using the ICD-10 code "F50". We highlighted the 10 most common "principal" diagnoses based on the organ system involved and the 10 most specific "principal" diagnoses for all ED visits by patients with any diagnosis of eating disorder. We then highlighted baseline characteristics of ED visits with a "principal" diagnosis of an eating disorder.

Results

There were a total of 56,901 ED visits for patients with eating disorders in 2018. Among these, 7,979 had an eating disorder as the "principal" diagnosis. Patients who visited the ED principally for eating disorders were more likely to be young females and came from higher-income households; about a third were admitted with 22.1 million U.S. dollars in aggregate ED charges. Mental disorders, and injuries and poisoning were the most common principal diagnosis by organ system categories, while eating disorders, major depression disorder (MDD), hypokalemia, and dehydration are common specific reasons for ED visits among patients with eating disorders.

Conclusions

Eating disorders, and its medical complications and psychiatric comorbidities such as MDD are common reasons for ED visits among patients with eating disorders. Management of the underlying eating disorder and their psychiatric comorbidities through a multidisciplinary approach in the outpatient setting is invaluable in reducing ED utilization by these patients.

## Introduction

Eating disorders are debilitating, potentially deadly, and expensive psychiatric disorders that significantly impair one's physical health and psychosocial functioning. Altered attitudes toward body shape, weight, and eating all play key roles in the origin and maintenance of these disorders [1]. Although anorexia nervosa and bulimia nervosa are the two specified eating disorders according to the Diagnostic and Statistical Manual of Mental Disorders Fourth Edition (DSM-IV), the most common eating disorder diagnosis is the category “eating disorder not otherwise specified” [2]. There has been an increase in the incidence rate of eating disorders among the high-risk group of 15- to 19-year-old girls [2]. According to large United States studies, the lifetime prevalence of anorexia nervosa is 0.9% among adult females and 0.3% among males, while bulimia nervosa has a lifetime prevalence of 0.9% to 1.5 % among women and 0.1% to 0.5% among men [2]. Eating disorders have the highest mortality among mental health disorders, with high rates of medical complications such as hypoglycemic attacks, electrolyte, and cardiac abnormalities [3]. Young persons with eating disorders tend to present to the emergency department (ED) at a rate significantly more often than those without eating disorders [3].

However, there remains a scarcity of national U.S. data on reasons for presentation and ED utilization of patients with eating disorders. In this study, our aim was to outline the most common reasons for which patients with eating disorder present to the ED. We also aimed to study baseline demographic characteristics of patients who presented to the ED with a “principal” diagnosis of eating disorders. We obtained data from the 2018 Nationwide Emergency Department Sample (NEDS) to answer these clinically relevant questions.

## Materials and methods

Data source

NEDS is the biggest ED database in the United States. NEDS provides an estimate of visits to ED owned by hospitals at a national level. The NEDS was created and is maintained by the Agency for Healthcare Research and Quality. The NEDS contains about 30 million unweighted ED visits, which is roughly estimated to be about 145 million ED visits each year. Although each visit in NEDS 2018 can have only one "principal" diagnosis, a maximum of 34 "secondary" diagnoses can be imputed. The "principal" diagnosis is the main reason for the ED visit, whereas any other diagnoses other than the "principal" diagnosis are "secondary" diagnoses. Secondary diagnoses are not confounding variables since they are not the reasons for the ED visits.

Inclusion criteria

We abstracted data for all ED visits with either a "principal" or "secondary" diagnosis of eating disorder, using the International Classification of Diseases (ICD)-10 code "F50".

Statistical analysis

STATA Version 16 (StataCorp, College Station, TX) was used for our analysis. We used a “ranking command” to outline the 10 most common "principal" discharge diagnoses based on the organ system involved, and the 10 most specific "principal" discharge diagnoses for all ED visits by patients with either a "principal" or "secondary" diagnosis of eating disorders. We then proceeded to outline baseline sociodemographic characteristics of ED visits with a "principal" diagnosis of eating disorders [4].

IRB approval

Since NEDS contains depersonalized publicly available data, institutional board review (IRB) approval was not sought.

## Results

There were a total of 56,901 ED visits for patients with eating disorders in 2018. Among these, 7,979 had an eating disorder as the principal diagnosis. Among ED visits principally for eating disorders, 80.6% were females with a mean age of 23.9 years, with 47.4% pediatric patients < 18 years (Table 1).

**Table 1:** Baseline characteristics and most common reasons for ED visits of patients with eating disorders in the United States in 2018 *Emergency department visits with eating disorder as the “principal” diagnosis, **Emergency department visits with “any” diagnosis of eating disorder, i.e., either a “principal” or “secondary’’ diagnosis of eating disorder. ED: Emergency department; charge: charge for emergency department visit; USD: United States dollars; adults: 18 years and above; pediatrics: less than 18 years; income: median household income national quartile for patient’s Zip code; SNF: skilled nursing facility; ICF: intermediate care facility, MDD; major depressive disorder; not elsewhere specified:  symptoms, signs, abnormal results of clinical or other investigative procedures, and ill-defined conditions regarding which no diagnosis classifiable elsewhere is recorded. These are less well-defined conditions and symptoms that, without the necessary study of the case to establish a final diagnosis, point perhaps equally to two or more diseases or to two or more systems of the body.

Baseline characteristics of ED visits for eating disorder* (n=7,979)	
Variables	
Mean age, years	23.9
Adults, %	52.6
Pediatric, %	47.4
Female, %	80.6
Mean charge, USD	3283
Aggregate charge, million USD	22.1
Charlson comorbidity index score, %	
0	84.9
1	11.3
2	2.3
3	1.5
Income, quartile	
0-25	21
26-50	23.3
51-75	22.7
76-100	33.1
Insurance, %	
Medicare	11.1
Medicaid	36
Private	47.2
Self-pay	5.8
Hospital location, %	
Rural	8.5
Metropolitan	91.5
Hospital teaching status, %	
Non-teaching	22.7
Teaching	77.3
Weekend, %	20.2
Region of hospital, %	
Northeast	27.5
Midwest	17.8
South	36.3
West	18.4
Discharge quarter, %	
First quarter	25.9
Second quarter	23
Third quarter	26.5
Fourth quarter	24.6
Disposition, %	
Routine	58
Transfer to short-term hospital	1.2
Transfer to other facility (including SNF, ICF, and others)	4.8
Against medical advice	1.3
Admitted	34.7
Most common reasons for all ED visit by patients with bipolar disorder ** (n=56,901)	
By organ system categories	n (%)
Mental, behavioral, and neurodevelopmental disorders	19,840 (34.9)
Not elsewhere specified	8704 (15.3)
Injuries and poisoning	5220 (9.2)
Endocrine	5109 (9)
Digestive system	4153 (7.3)
Respiratory	3107 (5.5)
Genitourinary	2230 (3.9)
Infection	2124 (3.7)
Cardiovascular	1318 (2.3)
Hematologic and Neoplasm	1202 (2.1)
By specific principal diagnoses	n (%)
Eating disorder, unspecified	2058 (3.6)
MDD, recurrent, severe without psychotic features	1936 (3.4)
MDD, single, unspecified	1540 (2.7)
Other unspecified eating disorder	1530 (2.7)
Anorexia nervosa, unspecified	1495 (2.6)
Hypokalemia	1445 (2.5)
Anorexia nervosa, restrictive type	1292 (2.3)
Dehydration	995 (1.7)
Sepsis, unspecified organism	966 (1.7)
Bulimia nervosa	896 (1.6)

These patients were more likely to have minimal comorbidity burden, come from higher income households, have Medicaid, are privately insured, and present to metropolitan and teaching hospitals in the south. About a third (34.7%) of these patients were admitted. Aggregate charge incurred for these ED visits was 22.1 million U.S. dollars. Excluding the category "not elsewhere specified", mental, behavioral, and neurodevelopmental disorders, and injuries and poisoning were the most common principal diagnosis by organ system categories for patients with eating disorders who presented to the ED. Different types of eating disorders, major depressive disorder (MDD), hypokalemia, dehydration, and sepsis were common specific principal diagnoses for ED presentation for these patients.

## Discussion

In our study, patients presenting to the ED due to eating disorders were predominantly young females. Eating disorders are predominantly found in adolescent and young adult women. The lifetime prevalence of anorexia nervosa in females ranges from 1.2% to 2.2%, while the prevalence in males is 10 times lower [5]. We found that these patients had minimal comorbidities likely due to their young age. We found that patients who presented due to eating disorders were more likely to come from higher-income households. Studies conducted in the 1960s and 1970s correlated eating disorders with higher socioeconomic status (SES); however, recent studies using health questionnaires distributed to large heterogeneous populations have shown that eating disorders equally affect all people, regardless of SES [6]. A more recent systematic review on eating disorders and SES did not find any evidence of a relationship between eating disorders and higher SES [7]. The previously believed misconception that eating disorder is more common among patients of higher SES may lead to disparities in the identification and diagnosis of eating disorders among patients of lower SES and may explain our findings [7].

Eating disorders are associated with the highest mortality among psychiatric conditions and medical complications such as electrolyte disturbances, cardiovascular complications, and refeeding syndrome. These complications can necessitate admission for management and closer monitoring when these patients present to the ED [3,8,9]. This can explain why about a third of patients who presented to the ED due to eating disorders were admitted to the same hospital in our study. A previous study showed that the economic impact of eating disorders in the United States is significant [10]. This study, which estimated the one-year cost of eating disorders, showed that the total economic costs associated with these disorders were about 64.7 billion U.S. dollars in the fiscal year 2018-2019, equivalent to 11,808 U.S. dollars per affected person in the United States [10]. In our study, the aggregate charges for ED visits for patients with eating disorders were more than 22 million U.S. dollars.

Eating disorders have psychiatric comorbidities including MDD, post-traumatic stress disorder (PTSD), and substance use disorder [11]. The risk of suicide is also elevated in patients with eating disorders [12]. Mental, behavioral, and neurodevelopmental disorders, and injuries and poisoning were the most common ICD-10 code categories for reasons for ED presentation among patients with eating disorders in our study. We found that unspecified eating disorder was the most common reason for ED presentation among patients with eating disorders. MDD was the second most common reason for ED presentation in these patients. A strong association between MDD and eating disorder has been found in prior studies. A survey of a large sample of 838 patients with eating disorders found that 19.5% of these patients had co-existing MDD and 48.7% reported clinically significant depressive symptoms, with patients with anorexia binge-purging and bulimia nervosa being more likely to be diagnosed with MDD [13]. Electrolyte disturbances and dehydration were common reasons for ED presentation in these patients in our study. These are known complications of eating disorders that can cause ED visits and potential hospital admission [14,15].

Screening and management of eating disorders and their psychiatric comorbidities such as MDD are essential in reducing ED utilization of these patients. Primary care providers should screen for these conditions and timely refer these patients to a psychiatric health provider if needed for management. Strategies and policies to tackle the shortage of psychiatric health providers in the United States are important in ensuring that these patients receive appropriate outpatient care to reduce the need for ED utilization [16]. A multidisciplinary approach involving the primary care provider, psychiatric health provider, and patients' relations, especially among pediatric patients, should be employed in managing patients with eating disorders in the outpatient setting to reduce ED utilization [17].

We used a large nationally representative ED database, which is a strength of our study. However, this study has some limitations. The NEDS is a claims database based on ICD codes for billing; hence, errors due to coding may exist. Data on race, age of disease onset, and severity of disease are not available in NEDS 2018 database [4,18].

## Conclusions

Excluding the category "not elsewhere specified", mental, behavioral, and neurodevelopmental disorders, and injuries and poisoning were the most common principal diagnosis by organ system categories for ED presentation among patients with eating disorders. Eating disorders, MDD, hypokalemia, and dehydration are common specific reasons for ED visits among patients with eating disorders. Patients with eating disorders who present to the ED are predominantly young females, had minimal comorbidities, and were from higher-income households in the south, and about a third of them are admitted to the same hospital. Management of the underlying eating disorder and their psychiatric comorbidities through a multidisciplinary approach in the outpatient setting is invaluable in reducing ED utilization by these patients.
